# Current Evidence and Gaps for Outpatient Respiratory Tract Infection Diagnostics: A Call to Action

**DOI:** 10.1093/ofid/ofaf519

**Published:** 2025-10-22

**Authors:** Christen J Arena, Holly M Frost, Park Willis, Brian Raux, Minkey Wungwattana, Michael P Veve

**Affiliations:** Eugene Applebaum College of Pharmacy and Health Sciences, Wayne State University, Detroit, Michigan, USA; Department of Pharmacy, Henry Ford Hospital, Detroit, Michigan, USA; Division of Pediatric Infectious Diseases, Department of Pediatrics, University of Utah School of Medicine, Salt Lake City, Utah, USA; Office of Research, Intermountain Health, Murray, Utah, USA; Intermountain InstaCare, Intermountain Health, Salt Lake City, Utah, USA; bioMérieux, U.S. Medical Affairs, Salt Lake City, Utah, USA; bioMérieux, U.S. Medical Affairs, Salt Lake City, Utah, USA; Eugene Applebaum College of Pharmacy and Health Sciences, Wayne State University, Detroit, Michigan, USA; Department of Pharmacy, Henry Ford Hospital, Detroit, Michigan, USA

**Keywords:** ambulatory care, antimicrobial stewardship, outpatients, rapid diagnostics, upper respiratory tract infections

## Abstract

Antimicrobial stewardship programs (ASPs) are underrepresented in outpatient settings, where antibiotic use and overprescribing are common. Upper respiratory tract infections (URIs) account for 30% of outpatient antibiotic prescriptions, highlighting the need for enhanced ASP efforts. Rapid diagnostic testing (RDT) has important value in management of outpatient URIs, such as pharyngitis, and can lead to optimized prescribing practices and significant reductions in unnecessary antimicrobial use by facilitating accurate diagnoses. Implementation of outpatient RDTs is hindered by a lack of streamlined workflows, resources, and ASPs. These gaps often lead to suboptimal use of RDTs and misinterpretation of or failure to act on the results. Future RDT evaluations should include strategies to curtail unnecessary antibiotics and expand point-of-care testing (POCT) to additional settings to enhance antimicrobial stewardship. This paper reviews outpatient RDT initiatives, and specifically POCT, in URIs. Additionally, we highlight the need for more evidence demonstrating the impact on clinical outcomes and antibiotic prescribing with the implementation of RDTs.

Upper respiratory tract infections (URI) are among the most frequent reasons for outpatient encounters in the United States (US), often resulting in an excess of inappropriate antibiotic prescriptions [1]. While antimicrobial stewardship programs (ASPs) are traditionally based in acute care/inpatient settings, there is a growing need for the development of outpatient ASPs, where 80%–90% of antibiotic consumption occurs [1–3]. Since antibiotic resistance, adverse events, and unnecessary costs are fueled by antibiotic use, outpatient ASPs should prioritize interventions that optimize antibiotic prescribing practices for disease states commonly associated with antimicrobial overuse, including URIs [4].

Recent strategies have been introduced to guide and encourage judicious outpatient antibiotic prescribing, aiming to safeguard patient health and enhance clinical outcomes in outpatient settings [5, 6]. The Centers for Disease Control and Prevention (CDC) Core Elements of Outpatient Antibiotic Stewardship provides a framework of 4 core elements: (1) commitment, (2) action for policy and practice, (3) tracking and reporting, and (4) education and expertise [5]. This guidance document provides specific suggestions for outpatient ASPs to prioritize URIs for intervention, providing examples as conditions where “antibiotics are not indicated (eg, acute bronchitis, nonspecific upper respiratory infection, or viral pharyngitis)”, “antibiotics might be appropriate but are over-diagnosed, such as a condition that is diagnosed without fulfilling the diagnostic criteria (eg, diagnosing streptococcal pharyngitis and prescribing antibiotics without testing for group A Streptococcus)”, and “conditions for which watchful waiting or delayed prescribing is appropriate but underused (eg, acute otitis media or acute uncomplicated sinusitis)” [5]. Additionally, the Centers for Medicare and Medicaid Services (CMS) collaborate with the National Committee for Quality Assurance to collect Healthcare Effectiveness Data and Information Set measures (HEDIS®) that identify gaps in performance and establish targets for improvement, several of which specifically target URIs that account for more than 30% of all outpatient antibiotic prescriptions [6, 7]. There are 4 HEDIS® measures related to URI antimicrobial stewardship. These include appropriate testing for pharyngitis, appropriate treatment for URI, avoidance of antibiotic treatment for acute bronchitis/bronchiolitis, and antibiotic utilization for respiratory conditions [6]. These URI-focused initiatives are related to value-based reimbursement, pay-for-performance, patient satisfaction measures, and payor incentives and rewards [6].

Diagnostic tests are important tools that, when leveraged appropriately in URI management, can reduce antibiotic overuse, improve time to optimal antimicrobial therapy, and have important implications for improved patient quality factors [8]. Recent advancements in diagnostics have seen a shift from laboratory antigen-based to on-site rapid diagnostic tests (RDT), which can provide results in 5–40 minutes and with high specificity [8]. Current market diagnostics include antigen-based tests and molecular-based tests (nucleic acid amplification tests (NAAT) that are polymerase chain reaction (PCR) or other methods that can be single- or multiplex) [8]. An approach that is increasingly being utilized is to combine clinical prediction rules (CPR) with point-of-care testing (POCT) [9]. Point-of-care testing, combined with diagnostic CPR, can significantly enhance patient care by delivering real-time results that improve clinical decision-making and overall quality of care [9, 10]. However, successful implementation of any RDT may be constrained by clinic or emergency department (ED) staffing shortages, the need for specialized certification for test administration, and associated direct costs and reimbursement challenges [11].

This review describes contemporary outpatient antibiotic stewardship initiatives related to RDT, specifically POCT, and identifies gaps in the management of common outpatient URIs.

## CURRENT MANAGEMENT OF RESPIRATORY INFECTIONS IN THE OUTPATIENT SETTING

### Diagnosis

URIs remain largely clinically diagnosed based upon a patient's constellation of symptoms while considering their vital signs, potential exposure, past medical history, physical exam findings, and risk factors. Understanding that the root cause of most upper respiratory symptoms is viral, much of the job of the clinician is centered around identifying URIs, which are caused by or include a bacterial component, potentially benefiting from antibiotics, as well as those URIs caused by specific viruses (ie, largely influenza and novel SARS-CoV-2 viruses) with associated antiviral medications proven to be beneficial in higher risk cohorts of patients.

Compared to viral infections and their ability to cause inflammation and symptoms throughout the respiratory tract, bacterial infections are limited to the locations of the upper respiratory tract. Commonly diagnosed bacterial URIs include pharyngitis, acute otitis media (AOM), and acute bacterial rhinosinusitis. Of these diagnoses, only acute pharyngitis has a commonly used POCT to detect a potential bacterial infection, namely Group A *Streptococcus* (GAS) [12, 13]. Newer POCT PCR platforms detect GAS along with Group C and G [14]. Diagnosis of bacterial URIs is otherwise largely dependent on guideline recommendations by professional societies, along with specific diagnostic CPR [15, 16].

Bacterial causes of pharyngitis largely focus on GAS due to the potential sequelae of acute rheumatic fever and a small reduction in clinical symptoms for some patients who are treated with antibiotics. The Centor score, and a modified Centor score by McIsaac, is the most commonly referred to CPR to clinically assess for GAS infection in the USA [17]. The FeverPAIN score is a comparable, validated CPR used more frequently in the United Kingdom [18]. Studies evaluating the diagnostic accuracy of both CPRs have found limitations leading to increased use of antibiotics when a lower score threshold is set and missed opportunities to appropriately treat GAS infection when a higher score threshold is set, when compared to rapid antigen testing and culture or PCR [17, 18]. Because of this, these CPR scores are most often used to rule out those patients not requiring GAS testing, although they are not reliable enough to diagnose all infections without testing. These scoring systems also tend to be less accurate in pediatric patients compared with adult patients [19]. This represents an area for further studies to differentiate bacterial from viral causes of acute pharyngitis [20].

Diagnosis of AOM is based upon findings on clinical exam, including moderate to severe bulging of the tympanic membrane (TM). The 2013 American Academy of Pediatrics guideline on the diagnosis and management of AOM in children aged 6 months to 12 years is frequently cited as the primary evidence-based source for AOM, which recommends diagnosis be based upon moderate to severe bulging of the TM or new onset of otorrhea not due to otitis externa [21]. Most cases of AOM resolve without an antibiotic, and clinicians should carefully weigh the risks and benefits of antibiotic use based on individual patient characteristics, including age, severity of symptoms, and duration of symptoms. Although recognized as a cause of illness with potential sequelae, the burden of adult AOM is much lower than pediatric AOM, with little data [22].

Viral respiratory testing has become increasingly available in outpatient clinics. Desktop POCT analyzers provide accurate PCR diagnosis with relatively fast turnaround times [23–25]. Most POCT NAAT analyzers can test for influenza A/B, SARS-CoV-2, and respiratory syncytial virus (RSV) [8]. Expanded viral targets on upper respiratory POCT have significant potential antimicrobial stewardship value (ie, avoid unnecessary antibiotics in virus-positive cases). However, real-world studies are needed to confirm this potential in the outpatient setting.

## ANTIBIOTIC STEWARDSHIP OPPORTUNITIES IN THE MANAGEMENT OF ORIs

Given the high frequency of outpatient URI encounters, ASPs should focus on URIs as an optimization strategy. ASPs are essential to foster a culture that supports appropriate antibiotic use, where a selection of key practices include (1) appropriate patient education to manage patient expectations and reduce prescribing pressures, (2) utilize RDTs to quickly and accurately identify the suspected pathogen and ensure antibiotics are prescribed only when necessary, (3) implement regular audits and clinician feedback, and (4) leverage existing efforts to report antibiotic stewardship metrics. The following studies highlight outpatient ASP efforts aimed at reducing inappropriate antibiotic use in URIs through the utilization of these key practices.

### Behavioral, Education, and Electronic Health Record Initiatives

Dutcher and colleagues conducted a stepped-wedge cluster randomized trial within 30 primary care clinics to evaluate the impact of a provider-targeted intervention on antibiotic prescribing for respiratory tract diagnoses [26]. A one-time educational session on patient communication strategies and appropriate prescribing for ORIs was performed, followed by monthly electronic feedback to providers on their prescribing habits [26]. There were 127 324 and 58 431 unique office encounters included in the pre-intervention and intervention period, respectively. Antibiotic prescriptions significantly decreased from 35% (pre-intervention) to 23% (intervention), *P* < .001 [26].

Another study evaluated the success of a health system-wide, multifaceted ASP bundle aimed at reducing unnecessary antibiotic prescribing for URIs in the outpatient setting administered by the ASP team (at least one Infectious Diseases/ASP pharmacist and physician) [3]. The bundle consisted of provider education in primary care, urgent care, and ED, including symptomatic management handouts for patients, an electronic health record (EHR) clinical decision support tool, peer comparison reporting, and an EHR dashboard for provider self-auditing when an antibiotic was prescribed but considered unnecessary [3]. The authors concluded that the targeted outpatient ASP intervention reduced URI prescriptions by 48%, preventing approximately 7300 unnecessary antibiotic prescriptions [3].

While these interventions have demonstrated success, their external validity and long-term sustainability may be limited. Behavioral and educational strategies often rely on ongoing engagement, institutional support, and resource availability, which may vary across settings. Additionally, the Hawthorne effect (where providers alter behavior due to awareness of being observed) may influence short-term outcomes but not reflect sustained practice change. In contrast, RDT-based interventions may be less susceptible to these limitations, offering standardized, objective diagnostic data that can support clinical decision-making without requiring continuous behavioral reinforcement.

### Rapid Diagnostics and Point-of-care Testing for Outpatient URIs

Implementing RDT in outpatient settings may have the potential to enhance the effectiveness of ASP efforts by providing timely and accurate information for more informed antibiotic decision-making. Compared to traditional methods, RDTs offer the potential to identify infections more quickly and may help differentiate between bacterial and viral etiologies. These timely results can support more informed clinical decision-making. Rapid diagnostic testing may contribute to antimicrobial stewardship efforts by guiding appropriate therapy and potentially reducing unnecessary antibiotic use. The most utilized URI RDTs include rapid antigen-based tests, microorganism-specific NAAT, host biomarker-based tests, and multiplex NAAT panels [11, 27]. More and more, POCTs are receiving Clinical Laboratory Improvement Amendments (CLIA) waived status [11, 28], thereby increasing the use of POCTs in the outpatient setting while maintaining high sensitivity and specificity [28]. POCT has even become part of pay-for-performance metrics included as a HEDIS® measure component (ie, CWP; “appropriate testing for pharyngitis”) [6]. This measure evaluates the proportion of patients with a pharyngitis diagnosis who receive an antibiotic prescription and an associated GAS RDT [6]. It is important to recognize that extensive testing has limitations, including costs of tests and reimbursement challenges, constrained resources, and challenges in interpreting complex results.

A recent study in a low-resource setting evaluated the use of a rapid antigen POCT for SARS-CoV-2, influenza A/B, RSV, and adenovirus [29]. The authors concluded that patients who tested positive for influenza were significantly more likely to receive antiviral therapy (98% vs 39%, *P* < .001) and less antibiotic therapy (2% vs 29%, *P* < .001) compared to those who tested negative [29]. Another study compared prescribing variability in URIs by providers and found that pediatricians were less likely to prescribe antibiotics for pharyngitis without a positive test for GAS than other health providers (pediatricians, 15% (95% confidence interval [CI], 10–22), other providers, 29% [95% CI, 21–40], advanced practice providers, 27% [95% CI, 19–37]; *P* < .0001) [30]. Blaschke and colleagues examined the used of rapid influenza diagnostic tests (RIDT) on influenza management in US EDs utilizing the National Hospital Ambulatory Medical Care Survey dataset [31]. Rapid influenza diagnostic tests were performed in 4.2 million visits, and 42% of influenza diagnoses were made in association with a RIDT [31]. The authors concluded that RIDTs decreased ancillary tests (45% vs 53%) and antibiotic prescriptions (11% vs 23%; absolute difference [AD], 12%; 95% CI: 0–23) with more frequent use of influenza antivirals (56% vs 19%; AD, 37%; 95% CI: 22–52) than visits without an RIDT performed [31]. A large randomized controlled trial (RCT) evaluating the impact of rapid respiratory viral testing on antibiotic prescribing in pediatric EDs found no significant reduction in antibiotic use, despite test results being available within an hour [32]. These findings suggest that diagnostic information alone may be insufficient to alter prescribing behavior, particularly in time-constrained, high-pressure environments. Several limitations may have influenced the study outcomes. Clinicians may have initiated antibiotic therapy prior to receiving test results, and the diagnostic panels used did not detect bacterial pathogens, potentially prompting precautionary antibiotic use. Additionally, the study did not incorporate educational or behavioral interventions, which are known to enhance the effectiveness of diagnostic tools in modifying clinical practice. The authors also performed parental surveys, indicating support for rapid testing, especially when results were available within 20 minutes [32]. Overall, the authors concluded that the utility of rapid respiratory panel testing may depend on its integration with broader antimicrobial and diagnostic stewardship initiatives [32]. This article, thus, serves as a “call to action” for the need of studies that describe the impact of RIDTs on optimal antibiotic prescribing.

## POTENTIAL BARRIERS AND CHALLENGES FACING POINT-OF-CARE TESTING FOR ORIs

Key factors to evaluate before RDT implementation include patient testing volume, the complexities of integrating multiple testing platforms, reimbursement and coverage, and connectivity with POCT middleware [11, 28, 33]. Perhaps one of the largest barriers ASPs face reflects which test to use for specific populations, particularly when considering the operationalization of POCT and ensuring these results are actionable. The sustainability of these efforts is a valid concern, particularly for smaller ASPs that may lack the resources to implement comprehensive interventions [33]. It would be considered best practice for the ASP to implement a POCT by conducting a formal evaluation (ie, cross-section or other study design) to ensure the implementation has been effective and to identify any barriers to their use. These practical questions highlight known barriers and challenges in the outpatient setting where leveraging the EHR, or eventually artificial intelligence, represents opportunity.

The challenges of POCT testing can include the location where the testing will be performed and which personnel will administer and interpret the test results [28]. Time constraints, contamination risks, quality control management and competency training, regulations and interface with the EHR are potential challenges [34]. Another challenge is integrating POCT into outpatient provider workflows to ensure results are actionable. Interpretation of the results may be considered a barrier to proper management [34]. While positive results can expedite the initiation of therapy, it is also important to consider the potential harm of treating colonized patients and patients with prolonged viral shedding [11, 28, 34]. Triaging the next steps following a negative or indeterminate test result may require additional testing to rule out other transmissible infections that POCT may not detect [28].

Currently, manufacturers seek to have their testing classified as CLIA-waived by the Food and Drug Administration (FDA) to reach a larger market for patients and providers to have broader access. Though CLIA-waived tests are not subject to the stringent regulations of traditional clinical laboratories, they still have specific requirements that require adherence [28, 35, 36]. Lastly, it is important to consider the costs associated with implementing CLIA-waived and POCT that may be more expensive than laboratory-based testing. An important consideration in the adoption of rapid respiratory panel testing is the cost and reimbursement. Test prices can range widely depending on the platform and scope of the panel. For example, limited multiplex panels (eg, testing for 3–5 pathogens such as influenza A/B and RSV) may cost $100–$150, while broader panels detecting over a dozen pathogens can exceed $300–$500 per test [23, 24, 37, 38]. These costs are further influenced by whether the test is performed in-house or sent to a reference lab, and by the clinical setting (eg, outpatient vs ED). In outpatient settings, reimbursement remains limited, particularly for large multiplex panels, which are often not considered medically necessary unless specific criteria are met (eg, immunocompromised status or high risk for complications) [38]. These financial and policy-related barriers highlight the need for further evaluation of the clinical utility and cost-effectiveness of respiratory panel testing across diverse healthcare environments [23, 24, 37–39].

## CONCLUSIONS AND FUTURE DIRECTIONS

Enhancing outpatient antimicrobial stewardship for URIs represents a critical area for improving patient outcomes, reducing unnecessary antibiotic use, and mitigating the burden of antibiotic resistance. URIs represent the most common infectious disease states treated with antibiotics, while most do not reflect bacterial infection. Furthermore, they also have pay-for-performance implications that impact health systems. RDTs, and particularly POCT, have shown potential to enhance patient care in outpatient URIs, although implementation barriers are common [34]. To overcome these challenges, it is essential to supplement RDTs with robust, proactive stewardship practices (ie, audit and feedback). Stewardship programs should consider the following: perform routine audits, provide education on test interpretation, demonstrate successful implementation of CLIA-waived POCT in diverse settings, and streamline workflow processes.

While the landscape of RDTs used in outpatient settings has continued to evolve, there remains a need for pragmatic research to develop and evaluate implementation strategies, assess their real-world impact, and better understand associated costs and benefits. Such research can help build a more balanced evidence base to guide future practice. These findings may be valuable to other health systems operating across diverse outpatient settings (ie, EDs, urgent cares, and ambulatory clinics), which can often face unique implementation challenges and practice variations, including private practices and specialty consultants. It is often challenging for ASPs to identify which technology would provide the greatest patient benefit in a rapidly evolving landscape of various molecular platform testing. These data should also include results beyond timeliness of results and include healthcare resource utilization endpoints that matter to prescribers, patients, payors, and health system leadership. These could include quality of life outcomes, such as symptom resolution and return to normal daily living, or other qualitative results from the prescriber and patient perspective. The implementation of various RDT platforms would benefit from mixed-methods studies describing the above pontifications.
